# Tuberculosis, as a Disease of the Masses, and How to Combat It

**Published:** 1902-09

**Authors:** 


**Tuberculosis, as a Disease of the Masses, and How to Combat It.** By S. A. KNOPF, M.D. London: Rebman, Limited. 1902.

This essay, of 76 pages, received the international prize awarded by the International Congress to Combat Tuberculosis, which met at Berlin, May, 1899. Its motto was as follows:—To combat consumption as a disease of the masses successfully requires the combined action of a wise Government, well-trained physicians, and an intelligent people.

The English edition has been adapted by Dr. J. M. Barbour, of Swanage.

